# Online-Offline Teaching for Bio-Pharmaceutical Students During the COVID-19 Pandemic: The Case Study of Advanced Mathematics in Application-Oriented Universities of China

**DOI:** 10.3389/fpubh.2022.911117

**Published:** 2022-07-14

**Authors:** Weicai Peng, Shuchao Wang

**Affiliations:** School of Mathematics and Statistics, Chaohu University, Hefei, China

**Keywords:** pandemic, bio-pharmaceutical, application-oriented universities, mathematical modeling, outcome-based education

## Abstract

**Background:**

With the development of the COVID-19 pandemic, the importance of online teaching is becoming more and more prominent, especially for the basic advanced mathematics majoring in bio-pharmaceutical in colleges. However, the only online teaching model loses efficiency when facing the undergraduates in application-oriented universities.

**Purpose:**

How to improve the teaching quality of advanced mathematics has always been a concern because the mathematical abilities of students in application-oriented universities are not ideal. In this article, we develop a blending online-offline teaching model that combined online teaching and offline outcome-based education (OBE), as an alternative to traditional offline education.

**Methodology:**

The comparative analysis experiment is carried out to the two classes of undergraduates. The control group and the experimental group are, respectively, the 2020 class students and the 2021 class students majoring in bio-pharmaceutical. The experimental group students receive the combined teaching method, while the control group students receive the traditional offline education.

**Results:**

(1) From the comparative analysis, we can find that the students under the online-offline teaching model are more differentiated than those under the traditional offline education model. (2) The online-offline teaching model equipped with “case study + knowledge point + applications” process has achieved a good teaching effect in the author's university.

**Conclusion:**

The proposed teaching model can well stimulate students' interest in advanced mathematics learning and resonate with students through actual cases, thereby arousing students' autonomous learning drive and allowing them to apply what they have learned to professional fields.

## Introduction

Advanced mathematics is a very important general basic course for high school students, as well as undergraduates majoring in bio-pharmaceutical in colleges. It has the characteristics of high abstraction, strict logic, and wide application. Advanced mathematics teaching goal orientation to develop the students' thinking ability and application ability, enables the student to master and apply knowledge of advanced mathematics to questions, think, and solve problems, and subsequent related courses to lay the foundation for the students, help students better grasp professional knowledge, and apply talents. Under the outcome-based education concept, the promotion and application ability of advanced mathematics knowledge points is improved to a new level. In 2019, the Chinese Ministry of Science and Technology, the Chinese Ministry of Education, and the other four ministries issued a joint document on mathematics for the first time, calling for more attention to the subject. However, in application-oriented universities, the failure rate of advanced mathematics examinations is very high, and many students are even afraid of advanced mathematics. Faced with this dilemma, many schools [as shown in references (1–12) and their related references] have taken action to reform the teaching of advanced mathematics.

The COVID-19 outbreak has disrupted the offline face-to-face teaching model, and when all students must leave the classroom to prevent gathering and spreading the virus, online learning may be the only solution [as shown in references (13–32)]. In recent years, many universities have begun to promote online teaching models [as shown in references (33–35)]. Due to its convenience and directness, online live teaching is favored by many teachers of advanced mathematics in chemistry, medicine, etc. However, if we simply choose to teach advanced mathematics to bio-pharmaceutical students online, the teachers will not be able to fully grasp the learning status and understanding of bio-pharmaceutical students. Therefore, neither the traditional teacher-led blackboard teaching mode nor the simple online teaching mode is suitable for the teaching of advanced mathematics for bio-pharmaceutical students under the normal situation of epidemic prevention and control. How to improve the teaching effect of online live teaching of bio-pharmaceutical advanced mathematics under the situation of normal epidemic prevention and control has become an important research topic for teachers.

In combination with the course characteristics of advanced mathematics, years of teaching practice experience, and practical problems encountered in online live teaching during the COVID-19 pandemic, we develop a blending online-offline teaching model that combined online teaching and offline outcome-based education, as an alternative to traditional offline education. The comparative analysis experiment is carried out on the two classes of undergraduates. The control group and the experimental group are, respectively, the 2020 class students and the 2021 class students majoring in bio-pharmaceutical. The experimental group students receive the combined teaching method, while the control group students receive the traditional offline education. The comparative experiment involved two randomly selected teachers who taught advanced mathematics in an application-oriented university of Anhui, China. The undergraduates' scores are evaluated by the two teachers and submitted to the official student score management system at the end of each semester. All the score data, which are shown in Appendix Tables A1, A2, were collected from the official student score management system.

### The Current Situation of Advanced Mathematics Teaching for Bio-Pharmaceutical Undergraduates in Application-Oriented Universities

Advanced mathematics courses are offered in application-oriented universities all over the country, among which science and engineering are usually called advanced mathematics, economics and management are usually called calculus, and some liberal arts majors in colleges and universities also offer higher liberal arts mathematics. Advanced mathematics courses are generally opened in the freshman year and are usually the first basic course that students contact after entering the university, so they have great influence. In addition, advanced mathematics is an important course that affects students' admission and employment. Many graduate students, especially bio-pharmaceutical, engineering, and economic management majors, need to take part in advanced mathematics examinations. After investigation, it is found that advanced mathematics teaching in application-oriented universities is facing many difficulties.

(1) A poor mathematical foundation and a lack of understanding of the phenomenon. Taking the author's university as an example, among all the students offering advanced mathematics, the average score of the students in the college entrance examination is near the pass line (90/150), the students' overall mathematical foundation is weak, the abstract ability is insufficient, and the students lack the cognition of the applications of mathematics in real life. At the same time, most of the textbooks available for students' reference lack enough practical cases, almost all of them are abstract mathematical concepts, properties, theorems, and their proofs, and students lack an understanding of the origin and progression of these concepts. Therefore, it is difficult to be interested in advanced mathematics.

Due to this dilemma, the teachers of advanced mathematics in application-oriented universities need to dig deeply into the cases of combining knowledge points with phenomena, so that students can experience the practical significance of advanced mathematics. At the same time, teachers should guide students to understand the abstract concepts in advanced mathematics, then students can understand the mathematical principles behind the phenomenon and know why.

(2) The teaching mode is traditional, and the ability to promote and apply advanced mathematics cannot be exercised. At present, the teaching of advanced mathematics courses in most colleges and universities still adopts the “teacher-centered” classroom teaching mode that has not changed for many years, focusing on the installation of theoretical knowledge. With the training of students' autonomous learning abilities and mathematics application abilities, classroom teaching has become a teacher's “talk show.” The teaching methods are single-minded, mostly based on lectures; the classroom interaction effect is poor; students do not actively participate in discussions; the classroom atmosphere is dull; and students have no interest.

Although many application-oriented undergraduate universities began teaching reform, the traditional teaching of classroom teaching mode is still the main teaching mode of the vast majority of application-oriented undergraduate universities. The teaching content is only to pay attention to the systemic theory and knowledge of advanced mathematics knowledge, lack of practical knowledge and applied aspects. The teaching mode of advanced mathematics needs to be reformed urgently. To this end, many schools are actively promoting the flipped classroom teaching model, in which students play the leading role in the classroom. This model has achieved good teaching effects in many colleges and universities. However, for application-oriented universities, many students' cognitive ability and self-learning ability are not enough to quickly apply the knowledge they have learned to related fields, and the teaching effect of this mode is also greatly reduced.

Therefore, when facing the students of application-oriented universities, teachers need to help students refine and abstract the knowledge points to the theoretical level through various means after they have mastered the knowledge points. Guide students to understand the application of this knowledge point in many similar fields. This kind of multi-angle, multi-field application promotion is conducive to students' understanding and mastery of the concept, but also puts forward higher requirements for teachers' lesson preparation work.

**Figure 1:**
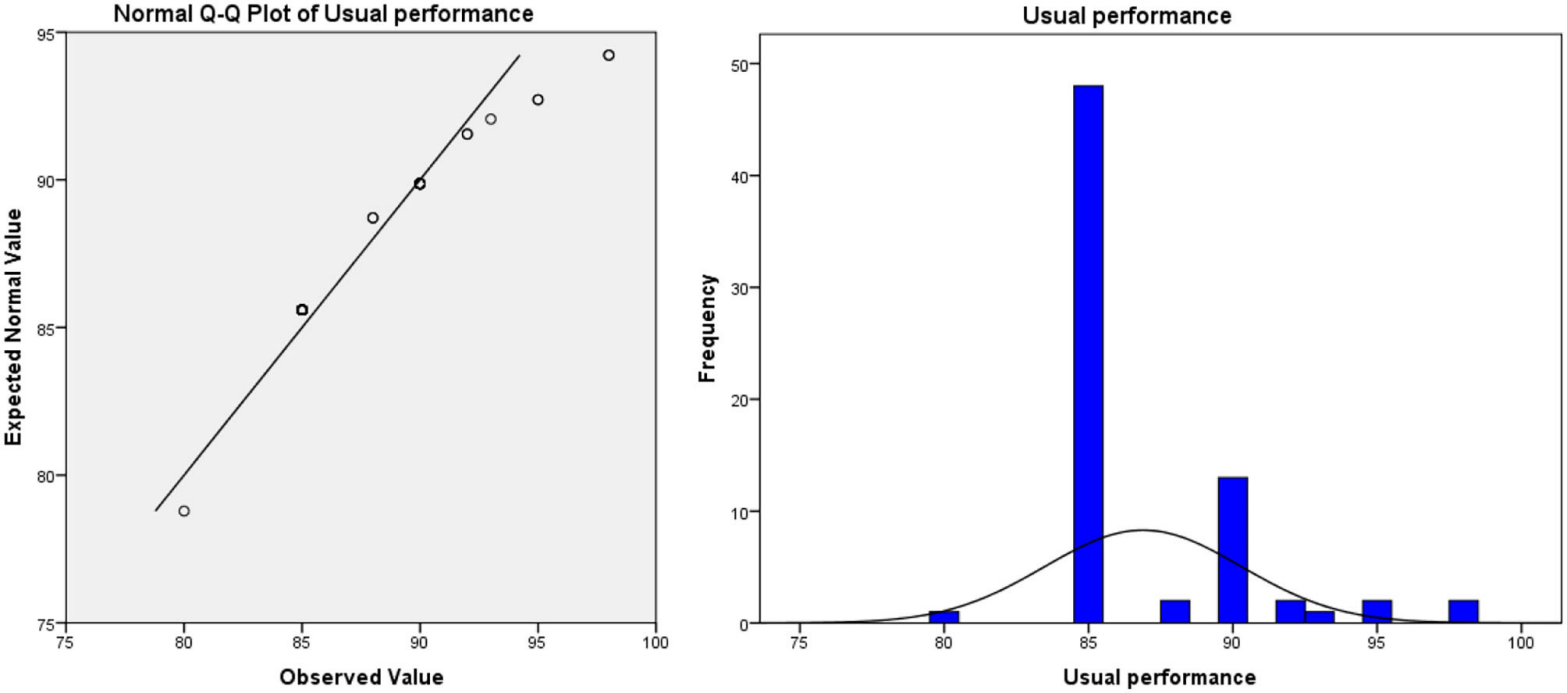
Usual performance of teacher-oriented graduates majoring in bio-pharmaceutical.

**Figure 2:**
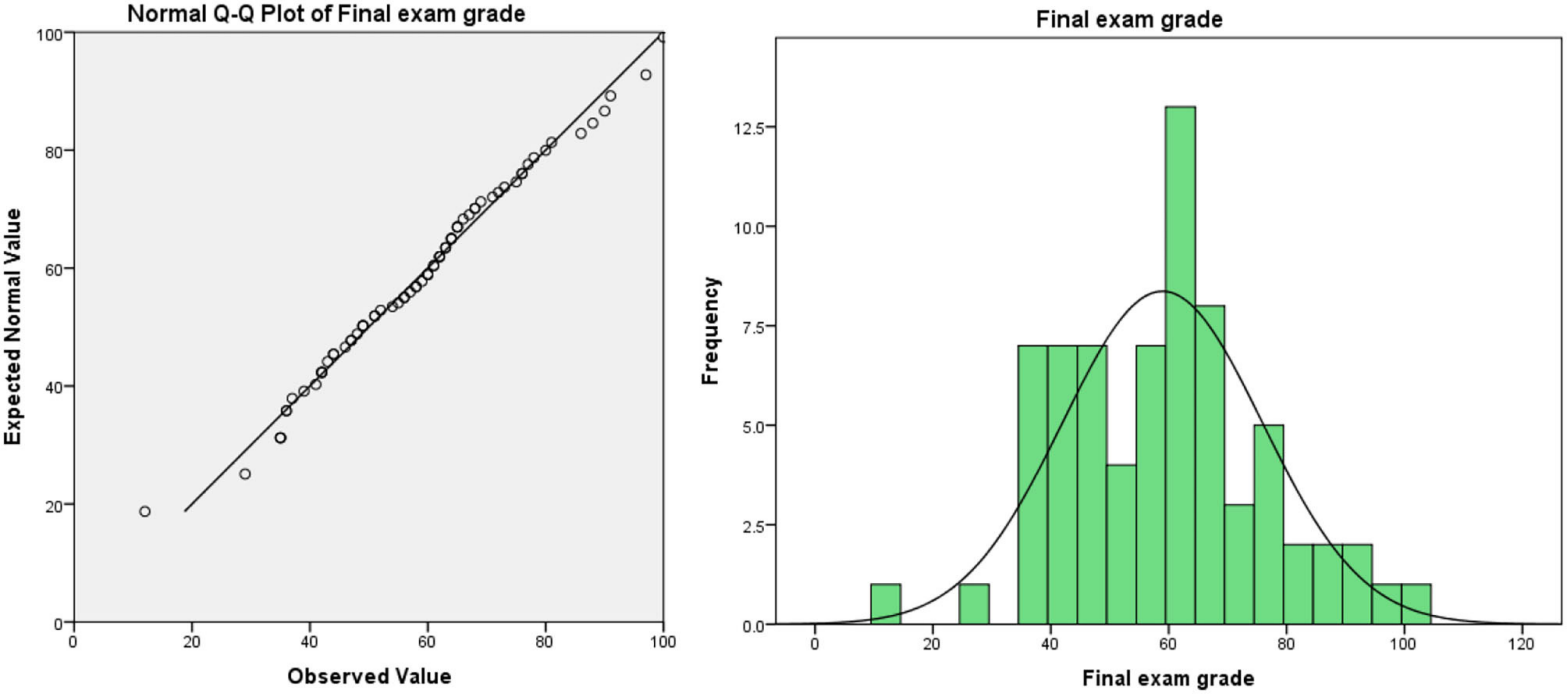
Final exam grade of teacher-oriented graduates majoring in bio-pharmaceutical.

**Figure 3:**
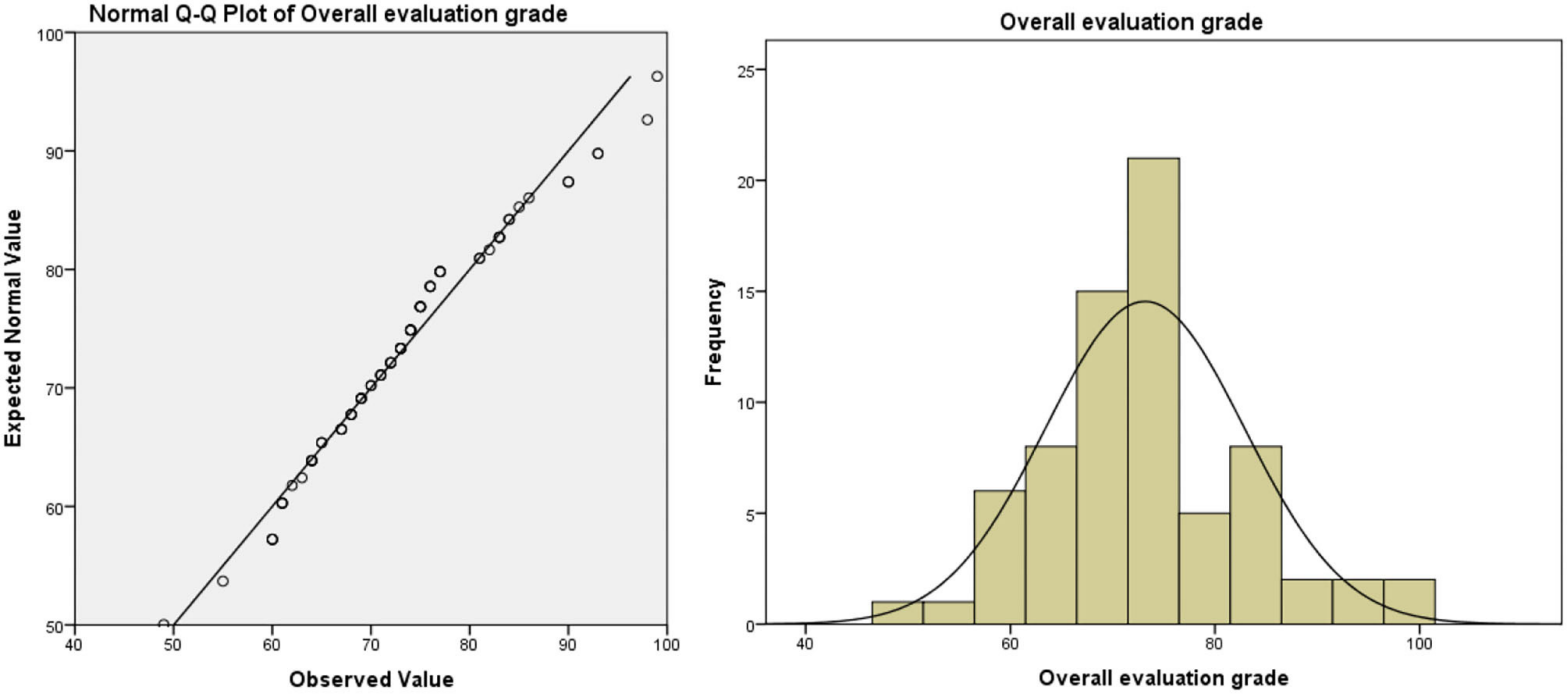
Overall evaluation grade of teacher-oriented graduates majoring in bio-pharmaceutical.

(3) The method of evaluating students' performance is not scientific enough. In the traditional teaching model, the usual assessment result is in the “usual performance × 50% + final exam score × 50% = overall evaluation score” mode. In this model, half of the scores need to be achieved in the final exam. Due to the proportion of ordinary results that are not attractive, leading to students usually lacking motivation. In the long run, this model will lead to some embarrassing phenomena: students “do not study in usual, but cram for exams,” teachers evaluate usual performance at will, which leads to the failure of the usual performance mechanism. Taking the author's university as an example, the final exam scores and overall evaluation scores of freshmen major in bio-pharmaceutical (the origin score data are shown in Appendix Table A1, sample size: 71, the teaching mode of these majors in that year was traditional teacher-oriented) are as follows (as shown in Table 1): the usual performance of the bio-pharmaceutical students is a mean of 86.89 and deviates significantly from the normal distribution (as shown in Figure 1). Meanwhile, the average score of the final exam is 58.93. The normal test shows that the distribution of the score deviates from the normal distribution, but the deviation degree is not serious (as shown in Figure 2). In terms of the distribution of the overall evaluation score, the average score is 73.15 (as shown in Figure 3). Based on this, we have reason to believe that the overall evaluation grade has not eliminated some interference factors, such as too much concentration of usual scores (that is, there is no differentiation of usual scores), which cannot achieve an effective incentive effect. This is also a common problem faced by the usual scores of application-oriented universities at present. It can be seen that under the traditional teacher-oriented teaching mode, there will be a great deviation between students' overall evaluation score and their actual mathematical analysis and application ability.

**Table 1:** Descriptive statistics of teacher-oriented graduates majoring in bio-pharmaceutical.

	***N***	**Minimum**	**Maximum**	**Mean**	**Std. deviation**
Final exam score	71	12	100	58.93	16.927
Usual performance	71	80	98	86.89	3.412
Overall evaluation score	71	49	99	73.15	9.737
Valid N (listwise)	71				

Therefore, the advanced mathematics teaching workers in application-oriented universities should, on the one hand, strengthen the supervision of students' learning processes so that the usual scores can reflect the students' usual learning situation. On the other hand, through the reform and innovation of teaching mode, stimulating the interest of students to improve the enthusiasm of participation, such that students can have a sense of gain and experience the fun of knowledge exploration.

## Online-Offline With “Case Study + Knowledge Point + Applications” Model

With the continuous development of the COVID-19 pandemic, the importance of online teaching is becoming more and more prominent, especially for the basic advanced mathematics majoring in bio-pharmaceutical in colleges. How to improve the teaching quality of advanced mathematics in application-oriented universities has always been a concern because the mathematics foundation of students in such universities is not ideal. This article proposes an online-offline advanced mathematics teaching mode of “case study+ knowledge point + applications” that attract students' attention through practical cases, guide students to have interest in knowledge points and further study and discussion. Under the guidance and refining of teachers, students can analyze, understand, and master knowledge points, and finally apply them to practical problems in their professional fields, and use the knowledge points to solve problems. Under this model, we introduce the case study online by using online sources; then concentrate on the knowledge point by offline face-to-face teaching; and finally, apply the knowledge to solve the practical problem through online examples or offline experiments.

The case teaching method originated from situational teaching cases at Harvard University in the United States and has gradually been widely recognized around the world. It refers to a teaching method that uses applied cases to create problem situations, takes students as the center, takes questions and cases as the guidance, and adopts heuristic teaching [as shown in reference (13)]. This mode simulates or reproduces some scenes in real life, lets the students bring themselves into the case scenario, carries on the new knowledge point study through the discussion, realizes from the case to the knowledge point study, namely, the “case study+ knowledge point + applications” mode. Based on this characteristic, the case teaching method adopted by application-oriented universities is helpful for students with a weak mathematical foundation to understand abstract mathematical knowledge more intuitively.

**Figure 4:**
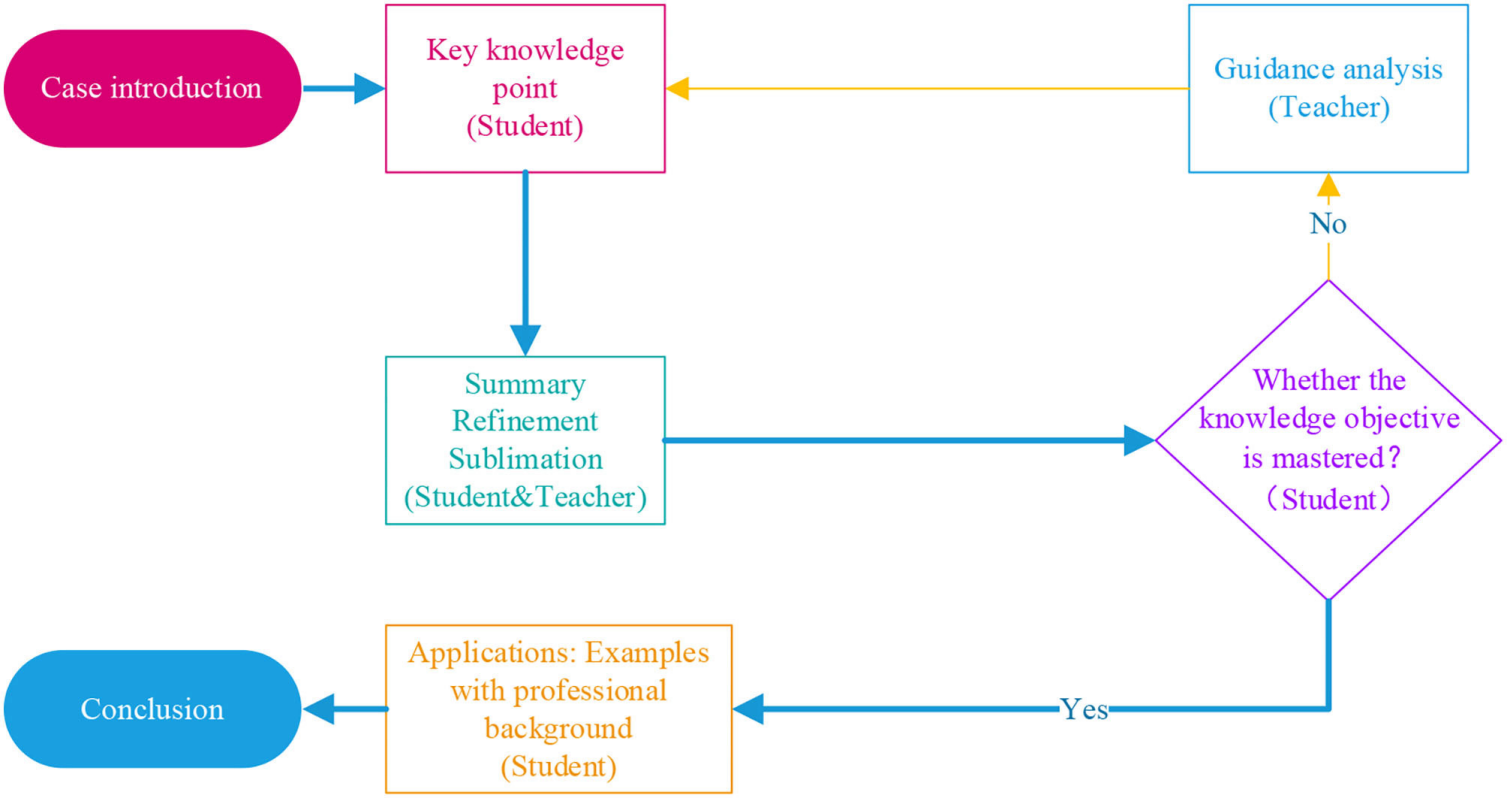
Teaching process under an online-offline with “case study+ knowledge point + applications” model.

For undergraduates in application-oriented universities, because the mathematics foundation is not solid enough and the traditional advanced mathematics teaching model is not efficient, many universities in China have begun to carry out teaching reform. After continuous exploration, based on the existing teaching experience in colleges and universities, this article puts forward an online-offline teaching mode with “case study + knowledge point + applications,” which is characterized by adding the link of “applications” based on “case + knowledge point” mode of the case teaching method. In the process of applications, on the one hand, students can consolidate the knowledge they have learned, on the other hand, they can promote the new knowledge they have learned to other fields and further realize the concrete understanding of abstract knowledge. The teaching process is shown in Figure 4.

### The Content of the Case Study

The content of the imported cases comes from what everyday college students have seen and heard, and it should be interesting; the imported cases should be “grounded,” and the cases shown are familiar to most students, preferably the kind that students know little about—students know this phenomenon, but do not know the reason behind this phenomenon. For example, ask students to measure the area of an irregular figure, or, if conditions permit, ask students to measure the area of a piece of land. This enables students to deeply appreciate the process of “partition, approximation, sum, and limit.” In this process, students can obtain an approximate area. How to obtain a more accurate area requires an in-depth understanding of the process of “taking the limit.” This is something that most students cannot master on their own and needs the guidance of teachers.

The introduction of cases should be in line with students' cognitive level—at their current level, students can find relevant mathematical problems through the case, and satisfy their curiosity and thirst for knowledge. To resonate with students, the case should be relevant to their existing life or learning experience. Appropriate teaching cases can help students easily enter the preset teaching environment, allowing them to gain new knowledge in pleasurable way. Case teaching can lead students to specific mathematical situations, more direct interaction between teachers and students, explaining the definition theorem through the teaching case, reducing the distance of mathematics and the living reality, thus realizing the distance between mathematics teaching and professional teaching, and allowing students in the study and research to understand the importance of advanced mathematics.

Rational use of multimedia and modern computer technology so that the case is more intuitive. Since mathematics is highly abstract, especially advanced mathematics [as shown in reference (10)], teachers can make teaching more concrete and vivid by using multimedia and modern computer technology in the teaching process. Through continuous training from concrete to abstract, students can better develop their abstract thinking. For example, the Fourier series is an important knowledge point in advanced mathematics, which has an important application in engineering. However, the Fourier series has a high degree of abstraction, so when showing the Fourier series and the Fourier transform, with the Fourier transform animation in the network resources (many video websites can view), it is very easy to understand the connotation of the Fourier series.

### Deep Understanding of Knowledge Points and Cultivating the Awareness of Mathematical Modeling

Students are exposed to new mathematical concepts, learn to distinguish the similarities and differences between the concept and other mathematical concepts, then become familiar with and master the mathematical concept, and finally become proficient in using the mathematical tools. The whole process needs to be achieved through continuous practice exercises. To a large extent, understanding the framework of the knowledge system, clarifying the path to solve problems, strengthening the cultivation of mathematical thinking, and strengthening the training of written expression skills need to be achieved through continuous problem-solving.

Teachers should actively play the role of “director” in the whole process of cultivating students to use the cited real case to discover and understand abstract concepts, establish mathematical models, solve practical problems, choose targeted topics, and carefully design and give lectures. For those question types with a certain degree of difficulty, the gradient should be designed in advance, step by step from simple to complex, from easy to difficult, and gradually increase the training intensity. It is an urgent problem to be solved in the application-oriented colleges and universities at present and in the future to strengthen the training of the basic problems of advanced mathematics for junior college students.

Through the practice of problem sets, the students' ability of analogy, the ability of analysis, the ability of induction and ability of abstraction, the ability of association, ability of deductive reasoning, ability of accurate calculation, the ability of learning, the ability to use mathematical software are trained. Through solving problems, it is necessary to cultivate students' ability to actively explore and grasp the essence of mathematical problems; skillfully describe mathematical problems with accurate, concise, and standardized mathematical language; find ways to solve problems from different perspectives;, be good at logical simplification and quantification of phenomena in real life; and establish mathematical models.

### Promotion and Application Reflect Professional Characteristics and Draw Inferences

After learning advanced mathematics knowledge points, students should not only be able to solve simple math problems but also be able to use it to solve problems related to their majors and apply them to their professional fields. For example, after understanding the concept of integral, students are further asked to explain the volume of space and variable force to do work. From area to volume is a natural extension, so most students can think of using the method of “partition, approximation, sum, and limit” for the volume problem of space, but most students will not think of applying this method to the problem of variable force work, which requires the teacher to point out. This puts forward higher requirements for the teaching of advanced mathematics: teachers should not only be proficient in advanced mathematics problems but also master the relevant background of the teaching profession and be able to skillfully transform the problems in the profession into necessary mathematical models and solve professional problems with mathematical models. For example, every year, the National College Students' Mathematical Contest in Modeling will involve physics, chemistry, economics, and other related topics, whose purpose is to test the students' ability to solve practical problems by comprehensive use of mathematical knowledge.

However, with the development of the times, more and more positions do not require a single major, and more and more graduates will turn to interdisciplinary fields. The core or key to these research fields is mathematics. The position also requires students to have a strong mathematical foundation. For example, traditional interdisciplinary subjects include biostatistics, sports statistics, and insurance actuarial, while emerging interdisciplinary subjects include big data, blockchain engineering, and artificial intelligence (AI). These majors all require high mathematical ability and are in great demand. To this end, teachers who teach advanced mathematics must keep up with the pace of the times, and while proficient in mathematics, they need to maintain an understanding of advanced technology.

Contemporary college students should be able to not only master the necessary advanced mathematics knowledge, but also be able to apply it in their majors, and gradually discover the application of this mathematical knowledge in other fields, to draw inferences from one case and learn by analogy. This requires college students to actively participate in mathematical modeling competitions, innovative projects, and teachers' scientific research projects. At the same time, students should participate in practical links, such as mathematical experiments, scientific computing, statistical experiments, and social investigations, and deeply realize that learning is useful, to experience a sense of gain from learning.

## Case Study: Sign-Preserving Property Application In A Kind Of Susceptible-Infective-Removal (Sir) Model

A sign-preserving property means that the sign of the value of a function satisfying certain conditions (such as, the existence or continuity of the limit) remains constant positive or constant negative in the local range. In this section, we use a case study to conduct the teaching process under the online-offline with the “case study+ knowledge point + applications” model.

### Learning Environment

This course is aimed at freshmen majoring in bio-pharmaceutical at an application-oriented university in Anhui Province in China. The average score of the students in the math college entrance examination is about 93 (the total score is 150). Before learning the sign-preserving content of function limit, students have completed the learning of limit content and learned the basic limit idea. However, it is difficult for students to rise from limited thinking to infinite thinking. So the teacher should make proper choices and organization to teach material content. This lesson, through the analysis of the case study, effectively breaks through the heavy and difficult point of this lesson—the concept and thought of the sign-preserving property.

In terms of teaching methods, based on the basic situation of students, this class is mainly based on the intuitive teaching method, supplemented by case teaching method and problem-driven method. Meanwhile, students' initiative in learning and enthusiasm in thinking about problems are fully mobilized, and the teacher's guidance is refined and sublimated. The use of citations can change the abstract into the concrete and deepen the understanding of the sign-preserving property.

### Learning Process

Stage 1: Case introduction. In the first 3 min of the course, a picture of the novel coronavirus is shown. Then, the students start to discuss the problem given by the teacher: under what conditions the pandemic will not spread.

Stage 2: Knowledge learning. The students have identified the problem and started to think about the solution. When thinking about the method to solve the problem, it prompts the students to use a kind of Susceptible-Infective-Removal (SIR) model. Assuming that the total number of people in the region remains the same for a period of time, at n-th day of the outbreak, the proportions of susceptible people, infective people, and removal people are denoted by *S*_*n*_, *I*_*n*_, and *R*_*n*_, respectively, where *S*_*n*_ + *I*_*n*_+ *R*_*n*_ = 1, *I*_*n*_ = *S*_0_ − (*lnS*_0_)/σ − (*S*_*n*_ − (*lnS*_0_)/σ), σ = exposure rate/recover rate, and *S*_*n*_ is monotone decreasing with S_n_ = S, (S < 1/σ). Under the guidance of the teacher, the students summarize and analyze the example, and summarize the method of solving the problem, which are applicable to the general similar problems. On this basis, the teacher summarizes the concept of sign-preserving property.

Stage 3: Generalization. Drawing inferences from one example and generalize. Finally, the content of this lesson is summarized: according to SIR model, to effectively stop the spread of the epidemic, we can (a) improve medical treatment and reduce exposure rate (making 1/σ bigger) and (b) herd immunity can be achieved by, for example, vaccination (reducing *S*_0_).

### Effect Analysis of Online-Offline With “Case Study + Knowledge Point + Applications”

**Figure 5:**
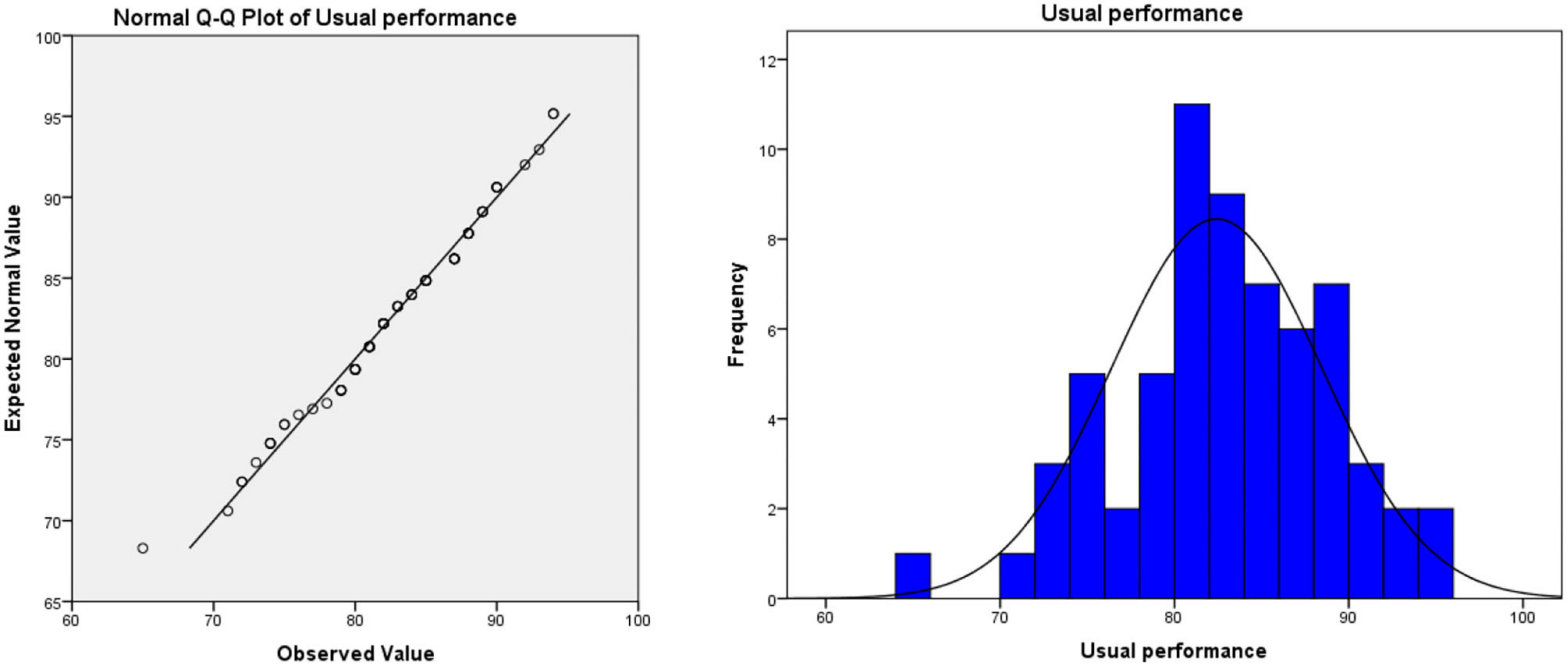
Usual performance of an online-offline teaching model.

**Figure 6:**
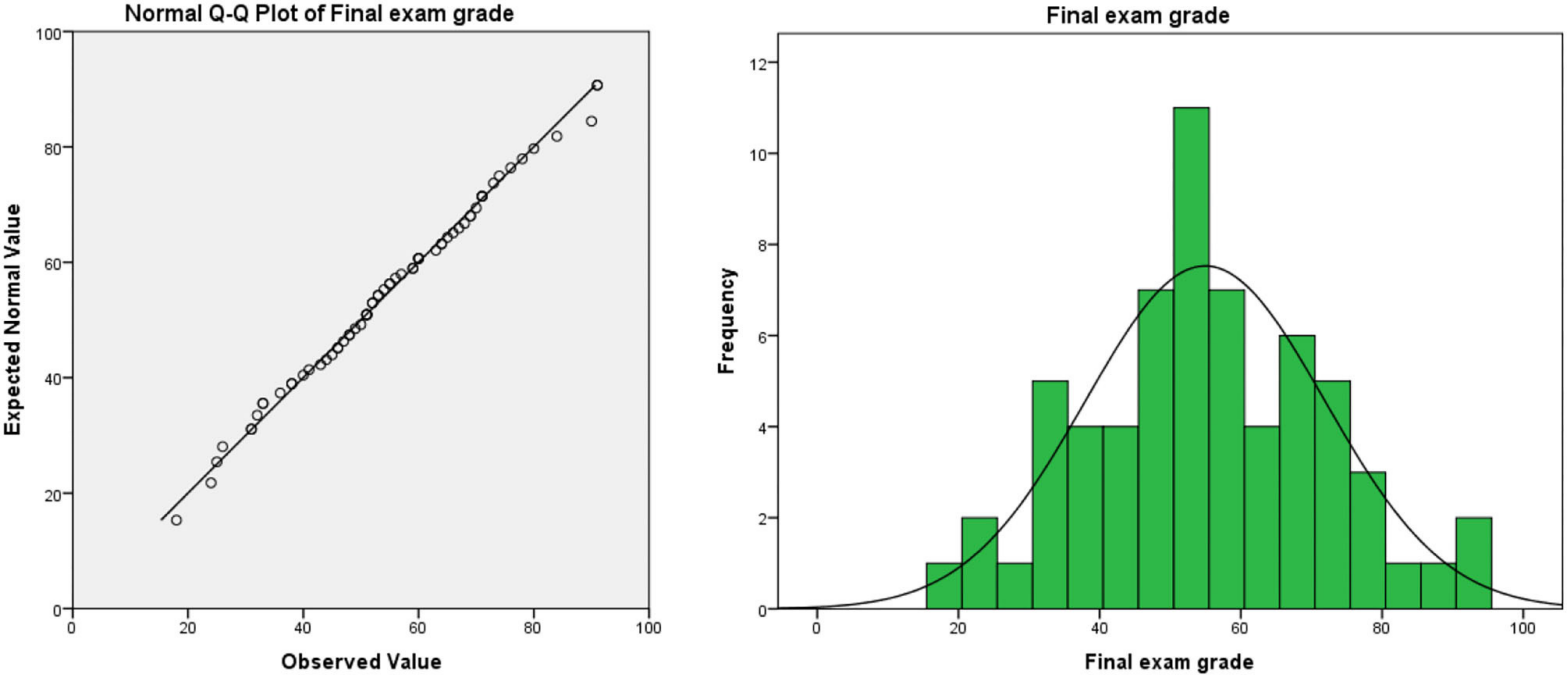
Final exam grade of an online-offline teaching model.

**Figure 7:**
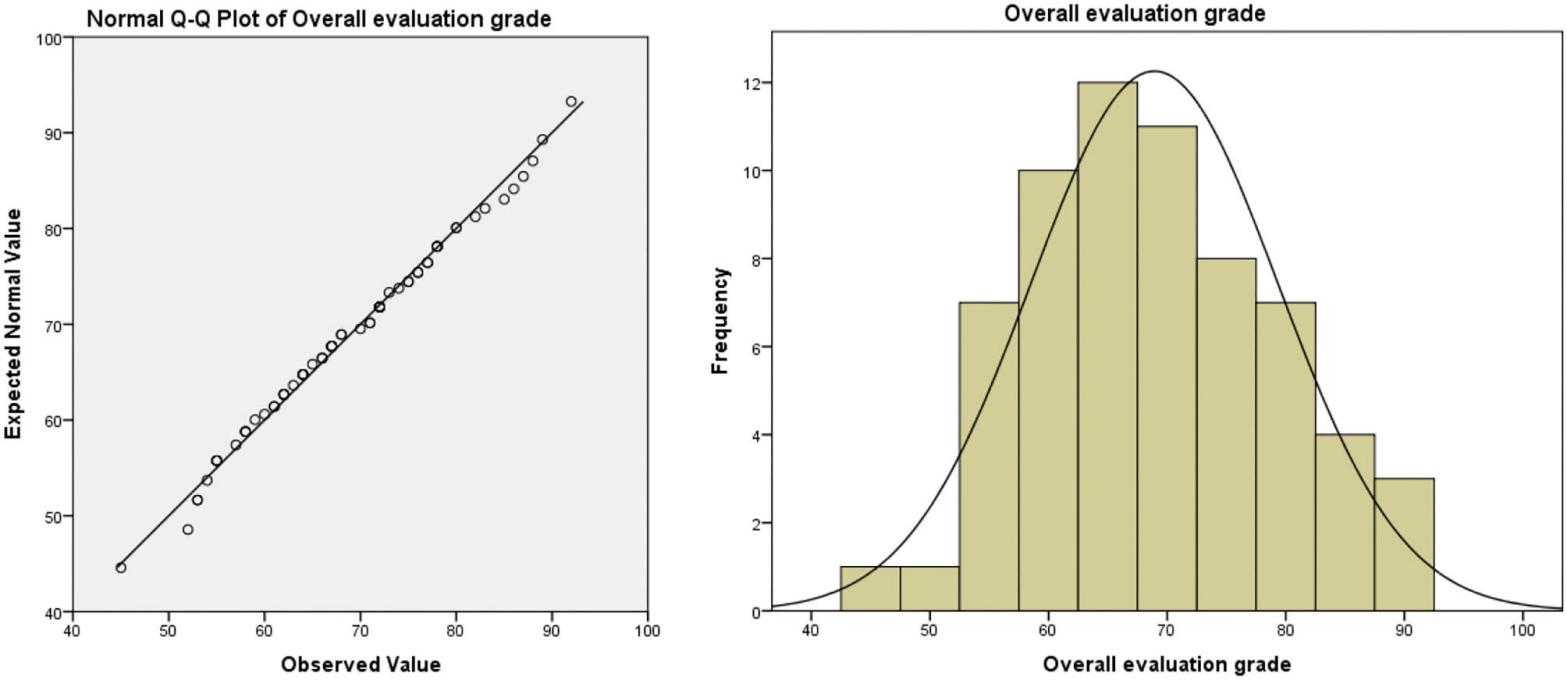
Overall evaluation grade of an online-offline teaching model.

The test group consisted of 64 first-year undergraduates majoring in bio-pharmaceutical in the author's university, adopting the teaching mode of online-offline with “case study + knowledge point + applications.” The control group consisted of 71 students from the previous grade of the same major, adopting the traditional teacher-oriented mode (the origin score data are shown in Appendix Table A2). The weights of ordinary and final grades are 0.5, respectively. The statistics descriptions and the test results of the experimental group scores are shown in Table 2 and Figures 5–7.

**Table 2:** Descriptive statistics of an online-offline teaching model.

	***N***	**Minimum**	**Maximum**	**Mean**	**Std. deviation**
Final exam grade	64	18	91	54.94	16.957
Usual performance	64	65	94	82.42	6.044
Overall evaluation grade	64	45	92	68.92	10.414
Valid N (listwise)	64				

Generally, from Figures 5–7, we can find that the usual score, final exam score, and overall evaluation score under the online-offline model with “case study + knowledge point + applications” conform to normal distribution. In terms of the average score, the class adopted the online-offline teaching mode of “case study + knowledge point + applications,” there was no significant difference between the classes with the traditional teaching model in the usual score, final exam score, and overall evaluation score. From the perspective of variance, the variance of the grades of the classes using the online-offline teaching mode of “case study + knowledge point + applications” (16.957, 6.044, and 10.414, respectively) was slightly higher than that of the classes using the traditional teaching mode (16.927, 3.412, and 9.737, respectively). These show that in the online-offline teaching mode of “case study + knowledge point + applications” grades are more differentiated.

## Conclusion

Due to the actual situation of students in application-oriented universities, the teaching reform of advanced mathematics is a very necessary and arduous task. Einstein once said that “interest is the best teacher.” This article aims to stimulate students' interest in learning, considers the online-offline teaching mode of “case study + knowledge point + applications,” and resonates with students through actual cases, thereby arousing students' autonomous learning drive, and applying what they have learned to professional fields.

However, there are thousands of teaching modes in practice. Advanced mathematics teachers in application-oriented universities should not only master teaching cases with a professional background but also keep pace with the times and apply knowledge points to advanced technologies through continuous learning.

## Data Availability Statement

The raw data supporting the conclusions of this article will be made available by the authors, without undue reservation.

## Author Contributions

WP: formal analysis, methodology, and writing original draft. SW: writing—review and editing. All authors have read and agreed to the published version of the manuscript.

## Funding

The Anhui Province Teaching Quality Project (2020mooc347, 2020jyxm1256, 2021jyxm1001); the University Outstanding Young Talents Project of Anhui Province (No. gxyq2021018, gxyq2019082); and the Teaching Quality Project of Chaohu University (ch19ylkc021).

## Conflict of Interest

The authors declare that the research was conducted in the absence of any commercial or financial relationships that could be construed as a potential conflict of interest.

## Publisher's Note

All claims expressed in this article are solely those of the authors and do not necessarily represent those of their affiliated organizations, or those of the publisher, the editors and the reviewers. Any product that may be evaluated in this article, or claim that may be made by its manufacturer, is not guaranteed or endorsed by the publisher.
