# Supplementary material for: Online-Offline Teaching for Bio-Pharmaceutical Students During the COVID-19 Pandemic: The Case Study of Advanced Mathematics in Application-Oriented Universities of China
Source: Front Public Health. 2022 Jul 14;10:911117. doi: 10.3389/fpubh.2022.911117 (PMC9330376; doi:10.3389/fpubh.2022.911117)
Supplement: Supplementary file 1 [file Table_1.pdf]

Appendix: The control and experimental group score data

Table A1 The control group score

No.	Final exam grade	Usual performance	Overall evaluation grade	No.	Final exam grade	Usual performance	Overall evaluation grade
**1**	12	85	49	**37**	60	85	73
**2**	52	85	69	**38**	64	85	75
**3**	36	85	61	**39**	36	85	61
**4**	35	85	60	**40**	46	92	69
**5**	68	85	77	**41**	65	85	75
**6**	63	85	74	**42**	51	85	68
**7**	42	85	64	**43**	44	85	65
**8**	75	90	83	**44**	58	85	72
**9**	48	88	68	**45**	49	85	67
**10**	61	85	73	**46**	55	85	70
**11**	61	85	73	**47**	78	90	84
**12**	72	90	81	**48**	65	85	75
**13**	59	85	72	**49**	66	85	76
**14**	49	85	67	**50**	62	85	74
**15**	97	98	98	**51**	41	85	63
**16**	42	85	64	**52**	54	85	70
**17**	37	85	61	**53**	60	85	73
**18**	60	88	74	**54**	64	85	75
**19**	39	85	62	**55**	69	85	77
**20**	76	90	83	**56**	67	85	76
**21**	47	90	69	**57**	57	85	71
**22**	71	90	81	**58**	47	90	69
**23**	90	95	93	**59**	62	85	74
**24**	91	95	93	**60**	65	85	75
**25**	35	85	60	**61**	73	90	82
**26**	44	85	65	**62**	64	85	75
**27**	80	90	85	**63**	63	85	74
**28**	29	80	55	**64**	100	98	99
**29**	51	85	68	**65**	35	85	60
**30**	68	85	77	**66**	81	90	86
**31**	56	85	71	**67**	76	90	83
**32**	58	85	72	**68**	62	90	76
**33**	56	85	71	**69**	86	93	90
**34**	88	92	90	**70**	77	90	84
**35**	49	85	67	**71**	43	85	64
**36**	43	85	64				

Table A2 The experimental group score

No.	Objective 1 (O1: weight 70%)			Objective 2 (O2: weight 30%)			Achievement degree of O1	Achievement degree of O2	Usual performance	Final exam score	Overall evaluation score
	Offline (15%)	Online (20%)	Final (35%)	Offline (5%)	Online (10%)	Final (15%)					
1	97.13	75.83	38.00	98.07	91.40	18.00	0.70	0.77	88	56	72
2	98.93	88.50	57.00	98.00	97.40	21.00	0.87	0.84	94	78	86
3	92.33	54.00	23.00	92.60	66.00	15.00	0.52	0.62	72	38	55
4	93.27	66.33	22.00	90.80	65.00	27.00	0.55	0.82	77	49	63
5	97.60	72.33	48.00	98.20	79.00	18.00	0.76	0.73	84	66	75
6	92.00	45.00	8.00	90.53	52.00	16.00	0.38	0.59	65	24	45
7	96.00	65.83	25.00	97.73	85.00	20.00	0.57	0.78	82	45	64
8	97.93	69.42	27.00	98.20	72.60	24.00	0.60	0.81	81	51	66
9	89.27	58.83	35.00	91.73	73.00	20.00	0.61	0.73	74	55	65
10	94.47	53.83	31.00	98.27	63.60	12.00	0.58	0.58	72	43	58
11	94.20	62.83	27.00	91.07	63.40	19.00	0.57	0.68	75	46	61
12	96.00	70.00	32.00	95.07	74.60	14.00	0.63	0.64	81	46	64
13	96.00	68.50	46.00	94.40	73.00	28.00	0.73	0.87	80	74	77
14	95.67	67.67	43.00	90.27	78.00	30.00	0.71	0.91	80	73	77
15	96.00	68.17	15.00	96.13	70.40	11.00	0.51	0.58	80	26	53
16	95.67	79.17	49.00	94.67	86.60	20.00	0.78	0.78	87	69	78
17	95.93	70.17	44.00	98.53	93.00	27.00	0.72	0.92	85	71	78
18	96.27	67.67	42.00	98.53	66.40	22.00	0.70	0.75	79	64	72
19	96.60	56.33	41.00	92.40	63.60	30.00	0.66	0.87	73	71	72
20	93.87	63.83	31.00	90.60	62.40	10.00	0.60	0.53	75	41	58
21	93.27	74.67	5.00	91.20	92.00	13.00	0.45	0.68	85	18	52
22	97.67	70.83	25.00	90.47	79.00	11.00	0.59	0.60	82	36	59
23	98.60	71.50	20.00	99.80	63.00	12.00	0.56	0.58	81	32	57
24	96.33	62.67	21.00	83.80	78.00	10.00	0.54	0.57	78	31	55
25	97.07	68.50	31.00	89.13	85.00	21.00	0.63	0.78	82	52	67
26	94.07	74.83	24.00	83.87	63.40	7.00	0.59	0.47	79	31	55
27	97.53	56.83	39.00	92.40	48.00	12.00	0.65	0.51	71	51	61
28	97.07	69.00	27.00	88.20	72.40	21.00	0.60	0.74	80	48	64
29	91.33	73.17	46.00	91.33	87.40	14.00	0.73	0.68	83	60	72
30	94.47	77.83	55.00	87.47	97.00	13.00	0.82	0.69	88	68	78
31	98.47	77.17	44.00	91.40	85.00	21.00	0.75	0.79	87	65	76
32	97.27	88.33	61.00	90.47	96.00	30.00	0.90	0.97	93	91	92
33	94.33	71.83	31.00	88.47	78.00	28.00	0.63	0.87	81	59	70
34	97.00	77.83	33.00	88.67	91.00	21.00	0.67	0.80	87	54	71
35	93.87	73.83	33.00	96.60	68.00	17.00	0.65	0.67	81	50	66
36	98.20	78.50	53.00	90.93	88.00	23.00	0.81	0.83	88	76	82
37	94.87	78.83	61.00	91.87	74.00	30.00	0.86	0.90	84	91	88
38	94.27	83.67	42.00	92.40	91.00	28.00	0.74	0.92	89	70	80
39	91.60	65.50	37.00	84.20	68.00	10.00	0.65	0.53	76	47	62
40	97.60	88.67	41.00	98.53	99.00	30.00	0.76	0.99	94	71	83
41	95.33	82.17	30.00	89.40	91.40	30.00	0.65	0.95	89	60	75
42	98.20	78.33	35.00	91.60	87.40	22.00	0.68	0.81	87	57	72
43	94.13	60.67	19.00	84.80	64.00	14.00	0.51	0.59	74	33	54
44	97.20	70.83	32.00	96.07	78.00	20.00	0.64	0.75	83	52	68
45	95.20	81.50	60.00	97.93	87.40	30.00	0.87	0.95	88	90	89
46	94.27	66.00	24.00	88.20	79.60	20.00	0.56	0.75	79	44	62
47	94.87	70.67	37.00	93.47	78.00	14.00	0.67	0.65	82	51	67
48	96.73	84.83	35.00	98.67	96.40	28.00	0.70	0.95	92	63	78
49	92.40	64.17	29.00	93.53	54.00	24.00	0.59	0.74	74	53	64
50	97.07	87.33	41.00	88.00	83.60	28.00	0.75	0.89	90	69	80
51	94.27	81.00	30.00	88.00	90.00	18.00	0.65	0.75	87	48	68
52	95.87	69.50	30.00	94.13	82.40	30.00	0.62	0.93	82	60	71
53	95.80	87.00	53.00	89.73	87.60	27.00	0.83	0.89	90	80	85
54	96.13	67.33	26.00	99.00	75.00	27.00	0.58	0.87	81	53	67
55	95.00	77.33	32.00	98.20	88.00	27.00	0.65	0.91	87	59	73
56	96.93	69.00	9.00	99.27	77.00	24.00	0.47	0.82	82	33	58
57	94.07	72.17	23.00	97.53	81.00	28.00	0.57	0.90	83	51	67
58	93.40	69.17	13.00	96.53	70.80	12.00	0.49	0.60	80	25	53
59	93.80	77.50	19.00	97.60	79.40	19.00	0.56	0.74	85	38	62
60	91.60	79.33	45.00	96.47	81.00	22.00	0.74	0.80	85	67	76
61	91.80	77.67	34.00	90.07	84.00	30.00	0.66	0.93	84	64	74
62	97.00	78.00	27.00	98.53	92.40	28.00	0.62	0.94	89	55	72
63	93.80	65.17	27.00	93.93	75.40	13.00	0.58	0.62	79	40	60
64	96.80	81.83	56.00	97.53	90.00	28.00	0.84	0.93	90	84	87
**Mean**	95.34	71.98	34.05	93.21	78.65	20.89	0.65	0.77	82	55	69
